# Screening, Diagnosis, and Treatment of Pediatric Ocular Diseases

**DOI:** 10.3390/children9121939

**Published:** 2022-12-10

**Authors:** Matthew Lam, Donny Suh

**Affiliations:** 1 Creighton University School of Medicine Phoenix Regional Campus, Phoenix, AZ 85012, USA; 2 Gavin Herbert Eye Institute, University of California Irvine, Irvine, CA 92697, USA

**Keywords:** vision screening, retinopathy of prematurity, retinoblastoma, strabismus, amblyopia, cataracts, glaucoma

## Abstract

Vision is an important aspect of a child’s quality of life and intellectual, social, and emotional development. Disruptions to vision during infancy and early childhood can cause lifelong vision impairment or blindness. However, early identification and treatment of eye disease can prevent loss of sight and its consequent long-term effects. Therefore, screening guidelines exist to guide physicians in detecting the most common threats to sight in the different stages of infancy and childhood. This review describes common causes of pediatric vision impairment, the recommended screening guidelines for diagnosing them, and current treatment modalities.

## 1. Introduction

Pediatric blindness is a life-altering condition that affects children worldwide. To address the then-estimated prevalence of 1.4 million irreversibly blind children, the World Health Organization considered control of blindness in children to be a high priority in its VISION 2020—The Right to Sight campaign that launched in 1999 [1]. More recent prevalence estimates as of 2020 range from 1.02–1.44 million depending on methodology, with 22.16 million estimated to have moderate-severe vision impairment and 46.60 million having mild vision impairment [2]. Preservation of vision is important as it plays a critical role during infancy and childhood, formative periods in which children learn to synthesize sensory information and engage with the world. Deficiencies in sight may impair early development and learning, resulting in lifelong intellectual, emotional, and social sequelae [3,4]. Many such issues can be avoided if deficits in vision can be identified and corrected early, but intervention becomes progressively difficult if disturbances to vision are allowed to persist.

To prevent loss of vision during infancy and childhood, early identification and treatment of ocular pathology are critical. Regular, systematic vision screening may assist in making early diagnoses of common causes of visual disturbances, many of which result in amblyopia and, ultimately, vision impairment. Amblyopia satisfies the World Health Organization guidelines for screening as it is a disease of significance that has an identifiable early phase, readily available diagnostic measures, and effective treatments [5]. This review seeks to assist pediatricians and other physicians with pediatric patient populations by outlining the most up-to-date screening guidelines and treatment options for common pediatric ocular pathology, especially those that frequently warrant management by pediatric ophthalmologists.

## 2. Vision Development and Early Screening

Pediatric vision screening is guided by the functional milestones of the visual system as it develops. At birth, a child’s visual system has not yet completed development and will have a visual acuity of approximately 20/400 [6]. Although it will be able to detect light and have an appropriate pupillary reflex [7], vision past its optimal viewing distance of 8–12 inches (20.3–30.4 cm) will be limited to blurred, gross shapes [8]. In the newborn nursery, pediatricians examine the eyes to elicit the red reflex and identify gross congenital defects such as cataracts, glaucoma, or infection. Detection of an abnormality or history of prematurity are indications for examination by an ophthalmologist [9]. Pediatricians continue to screen for similar abnormalities of the eyes during well-child physical exams [10,11].

By 2–4 months of age, infants have the ability to coordinate both eyes together and fixate on targets such as faces and moving objects. Intermittent strabismus may be noted until as late as 3–4 months but can be considered benign through this period [12]. Constant, large-angle strabismus (e.g., infantile esotropia or pathologic exotropia) may develop at this age; however, it is not benign. By 6 months, accommodation has completed development and stereopsis begins to progress [13].

Between the ages of 1 and 2 years, sight improves rapidly as sensory structures such as the optic nerves and visual cortex continue to myelinate and grow [14]. By ages 3–5, a child’s visual acuity reaches its adult level of 20/20, coinciding with the maturation of the fovea, which usually occurs near 4 years of age. At this point, robust visual acuity screening may be performed. Current guidelines by the American Academy of Pediatrics (AAP), American Academy of Ophthalmology (AAO), American Association for Pediatric Ophthalmology and Strabismus (AAPOS), and United States Preventive Services Task Force (USPSTF) uniformly recommend annual visual acuity screening from ages 3–5 years to identify issues such as amblyopia or its risk factors, including strabismus, anisometropia, and refractive errors (Table 1) [10,11,15,16]. In patients who are younger, preverbal, or have developmental delays, these screens are often conducted using instruments that can perform autorefraction or photoscreening, which do not require focused cooperation and feedback. In children who have developed the capacity to readily participate, subjective visual testing with HOTV or Lea symbols is preferred [10,11]. A cover test is also included in examinations to assess for refixation that would suggest strabismus. At 6 years and older, screening continues every other year. Visual acuity may be assessed using Snellen or Sloan letters, reflecting increasing literacy in older children.

The visual system reaches full maturity near the age of 10, at which point early-onset reversible vision impairment may no longer be able to be corrected. Children with amblyopia respond best to treatment before the age of 7 years, while children up to 13 years old typically respond less to treatment [17]. The age-dependent reversibility of some visual deficits in children highlights the importance of early screening and treatment.

## 3. Retinoblastoma

Retinoblastoma is a malignancy of the retina. It is the most common primary intraocular cancer in the pediatric population and affects approximately one in every 14,000–16,600 children, with roughly 95% of cases occurring before the age of 5 years [18,19,20]. Retinoblastoma classically arises due to mutations in both alleles of the retinoblastoma susceptibility gene (*RB1*), resulting in a lack of regulation at the G1-S checkpoint of the cell cycle and subsequent unchecked cellular proliferation. Patients who inherit a mutated gene and acquire the second mutation sporadically are deemed to have “heritable” or “germline” retinoblastoma (25–30% of cases), which typically presents bilaterally; those with two spontaneous mutations are considered to have “nonheritable” retinoblastoma (70–75% of cases), which usually presents unilaterally [21,22]. Untreated retinoblastoma fills and destroys the globe and can metastasize if it gains access to ocular vasculature such as the choroid plexus [22]. In high-income countries, 99% of cases are detected before metastasis, which correlates with survival rates as high as 97% or greater [23,24,25,26]. In contrast, 25% of cases are diagnosed after the onset of metastasis in low-income countries. Delayed diagnosis and treatment in these settings correlate with a survival rate of approximately 30%, demonstrating the importance of early screening and intervention [27,28].

Screening for retinoblastoma is a core component of routine vision screening beginning at birth, primarily with evaluation for the red reflex, as 50–60% of retinoblastoma cases present with leukocoria [29]. Other common presenting symptoms include strabismus (20%) and inflammation (5%). Leukocoria and strabismus, therefore, warrant urgent consultation by an ophthalmologist [9]. Further screening measures are recommended for children with a family history of retinoblastoma. For this high-risk population, recommendations made by the American Association of Ophthalmic Oncologists and Pathologists and endorsed by the AAPOS and AAP include serial dilated fundus examinations with or without anesthesia from birth until 7 years of age, with intervals dictated by risk [30]. The consensus panel also recommended genetic counseling and testing for mutation of *RB1* in all patients with personal or family history of retinoblastoma. Carriers are suggested to continue undergoing fundus examination indefinitely every 1–2 years after the age of 7 years, while those without mutation may discontinue after the age of 7 years provided they have remained asymptomatic. Diagnosis requires extended ophthalmoscopy under anesthesia, which classically reveals a soft, nodular, white or off-white mass(es) [31,32], augmented with A and B-scan ultrasound and MRI to characterize the mass and its extent of spread [22,33].

A wide variety of treatment modalities and strategies exists for retinoblastoma, though the guiding principle is consistent: preserve life, globe, and vision. Management is guided by the characteristics and classification of the tumor(s) [34,35,36], the exact details of which are beyond the scope of this more cursory review. Cryotherapy and laser photocoagulation are first-line local treatment options for low-risk tumors [22,37]. For larger tumors or those involving the macula, systemic chemotherapy may be used to first shrink the tumor to a size more amenable to focal therapy, a strategy known as “chemoreduction” [38,39,40]. For tumors of moderate to high risk, other chemotherapeutic options can be considered, including intra-arterial chemotherapy (IAC) and intravitreal chemotherapy. In IAC, chemotherapy is delivered through a cannula advanced to the ophthalmic artery with the goal of increasing drug concentration at the tumor site while reducing systemic exposure [41,42]. Treatment with IAC preserves the globe in 86% of early retinoblastoma cases, but the ocular salvage rate falls to 57% in advanced disease [43]. Intravitreal chemotherapy, in which chemotherapy is injected into the vitreous, is most commonly used to treat vitreous seeds refractory to IAC or systemic chemotherapy. It achieves seed control in 95% of cases, with an ocular salvage rate of 90.4% [44]. The risk of tumor dissemination due to intravitreal penetration has been found to be negligible, allowing intravitreal chemotherapy to be more frequently used in conjunction with IAC [45]. Compared to IAC alone, the combination results in a shorter time to regression, fewer recurrences, and an increased globe salvage rate [46,47]. In cases of retinoblastoma that are refractory to these globe-conserving measures and have poor visual potential, and in cases of large, advanced tumors that also have impaired vision potential, enucleation is indicated [22,37]. Systemic chemotherapy is sometimes used as an adjuvant treatment for cases that have extended past the globe and pose a metastatic threat [48]. Finally, systemic chemotherapy is also used for metastatic disease, sometimes as one component of intense multimodal therapy [49,50].

## 4. Retinopathy of Prematurity

Retinopathy of prematurity is a common disorder of ocular vascular development in premature infants. Previously the top cause of blindness in children in the United States, its prevalence in industrialized countries has fallen due to the widespread implementation of screening and treatment. However, it remains a leading cause of blindness worldwide [51,52]. Although prematurity is often defined as birth occurring prior to a gestational age of 37 weeks, retinopathy of prematurity is not typically observed in children born at or after 32 weeks of gestational age in developed countries [53,54]. Estimates of the incidence of retinopathy of prematurity vary dramatically based on study location, methodology, and definition of prematurity, ranging from 20% (2017 study in the United States) to 73% (2009 study in Sweden) [55,56,57]. Pathogenesis begins with an initial phase of premature cessation of ocular vascular development due to the relatively hyperoxic environment compared to the uterus. The resulting insufficient perfusion and increasing metabolic activity of the retina trigger the second stage in which excess vascular endothelial growth factor (VEGF) and erythropoietin (EPO) are secreted by the avascular retina. This triggers disorganized neovascularization that can proliferate into the vitreous, causing edema and hemorrhage due to leakage, fibrovascular tissue formation, and exertion of traction on the retina, which facilitates retinal detachment [55,58]. Therefore, the most prominent risk factors are the degree of prematurity or low birth weight, which exacerbate the immaturity of ocular vasculature, and supplemental oxygen, which is often required in premature neonates.

In the United States, screening for retinopathy of prematurity via binocular dilated indirect ophthalmoscopy is recommended for all infants born at or before 30 weeks gestational age or birth weight of 1500 g or less, as well as infants born after 30 weeks gestational age or birth weight of 1500–2000 g with additional risk factors as judged by a neonatologist [59]. Examination initially occurs at 31 weeks postmenstrual age (sum of gestational age at birth and chronologic age) for infants born at 22–26 weeks gestational age or at 4 weeks chronologic age for infants born at 27 weeks or later as determined by analysis of natural history data from the Multicenter Trial of Cryotherapy for Retinopathy of Prematurity and Light Reduction in ROP studies [60,61,62,63]. Follow-up examinations are indicated as frequently as less than one week out to as long as three weeks out based on the progression of vascularization and retinopathy within the retinopathy of prematurity zones as described in The International Classification of Retinopathy of Prematurity Revisited [59,64]. Repeat examinations are continued until vascularization is complete, the patient reaches 45 weeks postmenstrual age without the development of retinopathy, existing retinopathy regresses, or treatment is required [61,65,66].

Treatment is indicated when the criteria for Type 1 retinopathy of prematurity are met [67]. First-line treatments include anti-VEGF agents [68,69,70] and diode or argon laser photocoagulation [71,72]. Both anti-VEGF and laser therapies have similar efficacy in preventing progression and recurrence, but anti-VEGF treatment may be associated with a lower risk of structural adverse effects and high myopia compared to laser treatment [73,74,75]. Despite these advantages, anti-VEGF therapy, especially ranibizumab, requires closer monitoring due to the risk of reactivation or late recurrence, which usually requires subsequent laser treatment [76,77]. Cryotherapy, historically the only available treatment until the 1990s, is rarely used in settings where anti-VEGF or laser options are available due to their superiority [78,79].

## 5. Strabismus

Strabismus is a misalignment of the eyes, which compromises the ability to focus both eyes on the same target. This misalignment is caused by an imbalance between the extraocular rectus muscles. Strabismus is among the most common pediatric ocular pathologies, occurring in roughly 2–4% of children [80,81]. It may be present at birth, especially in premature or low birth weight deliveries [82,83,84], or be acquired during childhood, often in the setting of comorbid diseases such as vision deprivation, cataracts, or retinoblastoma [85,86]. Prolonged strabismus and the consequent visual discordance may disrupt the brain’s visual system as it develops, making it a leading cause of amblyopia. It may also be detrimental to the progression of binocular vision [87].

Due to its high incidence and debilitating sequelae if left untreated, pediatricians screen for strabismus during well-child checks. The cover test, in which the examiner covers the patient’s eyes sequentially and searches for refixation, is the most commonly performed examination technique. An asymmetric red reflex or Hirschberg’s corneal light reflex not symmetrically centered on the pupils are other findings that suggest the presence of strabismus [10,15,88]. More dramatic cases of strabismus, such as overt disconjugate gaze, may first be noted by parents or guardians.

Treatment of strabismus depends on multiple factors, including the type of strabismus (esodeviation vs. exodeviation), age of onset, and degree of misalignment (measured in prism diopters). Conservative, nonsurgical therapy may be considered in some cases; options include corrective eyeglasses, orthoptic exercises, prismatic correction, and miotic pharmacotherapy [89,90,91]. Conservative measures alone are sometimes indicated in children presenting with accommodative strabismus past the age of 1 year of age [92]. However, if large angle, constant non-accommodative strabismus is present prior to 6 months of age, surgery is indicated and should be performed as early as possible in order to maximize the development of stereopsis. Since stereopsis develops from birth to 2 years of age, strabismus during this time period disrupts the development of stereopsis. If the age of onset of strabismus is after 2 years of age, strabismus surgery has greater potential to restore stereopsis as the brain had previously developed stereopsis. Strabismus surgery is also indicated if conservative modalities fail or if strabismus is obvious and causes psychosocial issues [93]. Surgery is most often performed in cases of infantile strabismus (presenting within the first 6 months of life), nonaccommodative and partially accommodative esotropia, and cases with wide angles of deviation (esotropias greater than 15 prism diopters and exotropias greater than 20 diopters). Multiple surgical strategies exist to achieve ocular realignment by weakening, strengthening, or transposing extraocular muscles, and conservative treatment options are sometimes used to augment surgical correction. The goal of surgery is to reduce deviation to less than 10 prism diopters; achieving deviation of less than 4 prism diopters allows for the development of stereopsis [94,95]. 

It is imperative to differentiate between strabismus and similar appearing yet benign entities such as the aforementioned infantile physiological intermittent strabismus, which resolves no later than 3–4 months of age, and another phenomenon known as pseudostrabismus. Pseudostrabismus is the illusion of misaligned eyes due to particular facial features, most commonly a wide nasal bridge and prominent epicanthal folds, which can give the erroneous appearance of esotropia [96]. No management beyond reassurance and education of the family is required after ruling out true strabismus. Pseudostrabismus typically resolves without treatment as the child’s face matures with age, and while some reports have detailed a higher incidence of true strabismus in patients with pseudostrabismus, more recent studies have not been supportive of this risk [97,98]. 

## 6. Amblyopia

Amblyopia refers to impairment of vision processing secondary to disruptions to the development of the visual system due to anisometropia or high refractive error, strabismus, or obstruction of the visual axis [87,99]. Due to the commonplace nature of many of its risk factors, amblyopia is a very common cause of childhood vision impairment, with global prevalence estimated near 1–4% of all children [80,100,101]. Although amblyopia may present bilaterally, it is more commonly unilateral, resulting in the affected eye becoming “lazy” as the child relies more heavily on the unaffected eye. Consequently, the visual pathways associated with the affected eye do not develop adequately, leading to central visual deficits if not corrected before the visual system reaches maturity [102].

Amblyopia or its risk factors are evaluated by pediatricians during routine well-child checks. After first addressing underlying causes such as refractive errors and strabismus, treatment centers around penalization of the better eye to encourage the use of the weaker eye. The treatment modalities supported by the Pediatric Eye Disease Investigator Group studies continue to represent the gold standard. In addition to refractive correction, both physical patching of the strong eye and pharmacological penalization via atropine were found to be effective treatments [103,104,105]. Recent reviews support their continued use and demonstrate greater efficacy over alternative treatments such as optical penalization (Plano lens) [106,107,108]. 

## 7. Cataracts

Although cataracts are usually associated with aging and older patient populations, both congenital and acquired cataracts may be observed in pediatric patients. Infantile cataracts occur in approximately 3–14 of every 10,000 live births in developed countries and are responsible for 5–20% of pediatric blindness worldwide [109,110,111,112]. There are many etiologies of pediatric cataracts, including but not limited to infection such as intrauterine rubella or toxoplasmosis, trauma, metabolic disorders such as classic galactosemia, and inherited genetic tendency [113]. Cataracts are often detected by observation of the parents or guardians or by regular vision screening, revealing reduced visual acuity, red reflex asymmetry, or leukocoria [114,115].

As cataracts can completely obscure vision by opacifying the lens, removal of symptomatic cataracts is recommended to restore vision in the affected eye(s) and prevent amblyopia in younger patients. Patients with significant congenital cataracts should pursue surgery as soon as possible, ideally within the first 6 weeks of life [116,117]. Patients younger than 6 months will typically receive aphakic contact lenses to replace their natural lens rather than an intraocular lens, as is standard for older children and adults. The use of aphakic contact lenses has been proven to reduce complications and allows for more convenient modification of refractive power as the eye grows [118]. Secondary intraocular lens implantation is an elective procedure that may be performed later in childhood, adolescence, or adulthood per patient preference. Although the procedure has typically been reserved for those 1 year of age or older, recent research has suggested implantation can be done successfully as early as 7 months [117,119,120]. Following surgery, occlusion of the fellow eye is recommended to reduce the risk of amblyopia [121].

## 8. Glaucoma

Like cataracts, glaucoma is a condition of the eye primarily associated with advanced age. However, it too can affect pediatric patients, causing damage to the optic nerve that initially results in deficits of peripheral vision, then central vision and ultimately complete blindness if untreated [122]. Primary congenital glaucoma is the most common primary pediatric glaucoma. It is rare in industrialized countries, observed in one in every 10,000–30,000 live births [123,124], but can be as common as one in every 1250–2500 births in other populations and locations such as the Slovakian Roma and Saudi Arabia [125,126]. Pathogenesis involves abnormal development of the aqueous outflow track, particularly the trabecular meshwork or Schlemm canal.

Primary congenital glaucoma may present with some or all of the components of the classic triad of photophobia, epiphora, and blepharospasm due to corneal opacity or could simply present with buphthalmos (enlarged globe) [127]. Further examination may demonstrate corneal edema and cloudiness, corneal enlargement, and breaks in Descemet’s membrane, known as Haab striae. Diagnosis is made clinically with characteristic symptoms, physical exam findings, and usually, but not necessarily, measurement of intraocular pressure [128,129]. Although symptoms and manifestations suggestive of primary congenital glaucoma may be observed during routine eye exams, specific screening by measuring intraocular pressure is not performed outside of high-risk cases [11,130]. However, due to some genes, such as *CYP1B1*, being associated with familial manifestations of primary congenital glaucoma, genetic screening in high-risk populations may play a role in the future [131].

Surgery is the definitive treatment in primary congenital glaucoma. Goniotomy and trabeculotomy are first-line procedures that both have similar success rates of 80–90%, although trabeculotomy may be preferred in cases in which corneal hazing obscures the view of the angle [132,133]. In some cases, further surgery, such as tube shunting, is required. Pharmacotherapy is often used to supplement surgery but rarely replaces it; timolol is the first-line agent in pediatric glaucoma, but other options include carbonic anhydrase inhibitors, alpha-2 agonists (contraindicated in patients younger than 2 years of age), miotics, and prostaglandin analogs [122,134,135].

Although primary congenital glaucoma is the most common type of primary glaucoma in pediatric patients, primary glaucoma can also be observed later in childhood. Juvenile open-angle glaucoma can occur in children past the age of 3 years and resembles adult open-angle glaucoma but progresses more aggressively and requires surgery more frequently than its adult counterpart. Pediatric glaucoma also presents secondary to many other etiologies such as Sturge-Weber syndrome, Axenfeld-Rieger syndrome, Peters anomaly, aniridia, corticosteroids, and cataract surgery [136,137,138,139,140,141].

## 9. Conjunctivitis

Conjunctivitis, or “pinkeye”, is a common cause of eye redness resulting from inflammation of the bulbar and tarsal conjunctiva. It occurs most frequently in children below the age of 7 years and is most commonly caused by allergic reactions, viral or bacterial infections, and noninfectious irritants like smoke or fumes [142]. Diagnosis is made clinically with little role for culture or testing; concomitant clinical features can aid in the determination of the etiology, although the specificity of such features is often low [143]. Allergic conjunctivitis typically presents bilaterally with its characteristic pruritis; other features can include watery discharge and atopic symptoms such as congestion, sneezing, or wheezing. Viral conjunctivitis, most commonly caused by adenovirus [144], also usually involves watery discharge but can present unilaterally before the fellow eye is involved. Other viral prodromal features may be present. While bacterial conjunctivitis can also present unilaterally or bilaterally, it is best distinguished by the rapid onset of symptoms, including purulent drainage that returns minutes after wiping and can lead to the infected eye(s) being “stuck shut” upon waking. However, this adhesion is nonspecific and may also be observed in viral conjunctivitis [145]. The most common causative organisms are *Staphylococcus aureus*, *Streptococcus pneumoniae*, *Streptococcus epidermidis*, and *Moraxella catarrhalis*. *Haemophilus influenzae* historically also frequently caused bacterial conjunctivitis but has become less common due to widespread vaccination [146]. 

Treatment of conjunctivitis is directed toward the underlying etiology. Bacterial conjunctivitis can be self-limiting but can also be treated with antibiotic drops or ointments for 5–7 days to reduce the disease duration. Trimethoprim/polymyxin B, fluoroquinolones, and macrolides are effective treatments; fluoroquinolones are preferred in contact lens wearers for coverage of pseudomonas. Both viral and allergic conjunctivitis may be treated symptomatically with cold compresses and artificial tears. Allergic conjunctivitis may also be treated with topical antihistamines, mast cell inhibitors, or vasoconstrictors, though allergen avoidance is key in preventing symptoms [145,147]. The use of corticosteroids should be reserved for consulting ophthalmologists in specific cases of allergy and viral infection due to the risk of corneal damage, cataract, and glaucoma [148,149].

Infectious conjunctivitis, especially viral conjunctivitis, is highly contagious, and care must be taken to avoid spread. Bacteria and viruses are readily transmitted via direct contact or fomites, with up to 46% of patients with viral conjunctivitis having positive cultures grown from swabs of the hands [150]. Therefore, strict hand hygiene, such as hand washing, avoidance of touching one’s eyes, and abstinence from sharing personal items, are critical to prevention. In the United States, children are usually prohibited from returning to school or childcare services until 24 h after initiating treatment of conjunctivitis or resolution of discharge, although this is less effective for prevention of viral conjunctivitis given its estimated contagious period of 10–14 days [145,147,151].

In neonates, vertical transmission of infectious agents can cause ophthalmia neonatorum (neonatal conjunctivitis), most often due to infection by *Neisseria gonorrhoeae*, *Chlamydia trachomatis*, or herpes simplex virus (HSV). *Neisseria gonorrhoeae* and HSV infection can have serious complications, including ulceration and scarring of the cornea. Therefore, perinatal prophylaxis with 0.5% erythromycin ointment is recommended in the United States to prevent gonococcal conjunctivitis [152,153]. However, topical erythromycin is not an effective option for prophylaxis or treatment of chlamydial conjunctivitis. Treatment instead requires oral azithromycin to also treat potential concomitant infections of other organ systems, which are observed in over half of chlamydial conjunctivitis cases [146,152]. Treatment of HSV conjunctivitis involves oral acyclovir and topical 1% trifluridine, 0.1% idoxuridine, or 0.15% ganciclovir drops [154].

## 10. Lacrimal Duct Obstruction

Congenital blockage of the lacrimal duct is a common and benign problem observed in as many as 6% of neonates [155,156]. Tears are produced in the lacrimal gland found superolateral to the eye and coat the surface of the eye to maintain moisture, remove debris and microbes, and maintain clarity of the cornea. They then exit the surface of the eye through an opening of the medial eyelid known as the punctum and flow into the lacrimal canaliculi. From the canaliculi, they accumulate in the lacrimal sac and then drain into the lacrimal duct and ultimately into the nose. The most common type of lacrimal duct obstruction is dacryostenosis, which is usually caused by the persistence of a membrane at the distal end of the lacrimal duct that failed to regress during canalization [157]. If a proximal obstruction is also present, usually in the common canaliculus or junction between the common canaliculus and lacrimal sac, the blockage is considered a dacryocystocele [158]. 

Obstruction of the lacrimal duct presents with one or both eye(s) pooling and often overflowing with tears, resulting in them running down the cheek due to failure of tear drainage. Crusting and “mattering” of the eyelids and eyelashes are also common symptoms. In the case of dacryocystocele, a bluish mass superficial to the lacrimal sac may be present. Symptoms may be detected by caregivers or during routine eye examinations [11,15]. Diagnosis is clinical, though the dye disappearance test can be used in unclear cases, in which fluorescein is applied into the lower eyelid and monitored for disappearance within 5 min to indicate adequate tear drainage [159]. 

Congenital nasolacrimal duct obstruction is typically self-limited, with 78–96% of cases resolving spontaneously before the age of 12 months [160,161,162]. Cases that have not been resolved past 12 months are unlikely to do so spontaneously. Treatment begins with conservative measures such as lacrimal sac massages, which entail the application of moderate, downward pressure to the lacrimal sac with the goal of rupturing the imperforate membrane. Massages are performed multiple times daily until the resolution of symptoms [163]. Obstruction refractory to lacrimal sac massage requires surgical intervention, usually lacrimal duct probing, in which a small probe or cannula is advanced through the punctum and mechanically ruptures any obstructing membranes until reaching the site of tear drainage into the nose. Although congenital nasolacrimal duct obstruction often resolves spontaneously until 12 months, surgical probing can be performed as early as 6–10 months to eliminate symptoms earlier, avoid the need for general anesthesia, and potentially cause less lacrimal duct scarring [164,165].

Congenital nasolacrimal duct obstruction can be complicated by infection due to the proliferation of bacteria within the accumulated tears, which can present with purulent discharge. A 3–5 day course of topical antibiotics, typically fluoroquinolones, can be used to treat simple bacterial overgrowth [166]. However, purulent discharge in the presence of other symptoms indicative of infection, such as fever or erythema and tenderness in the location of the lacrimal sac, indicates a more serious complication known as dacryocystitis [167]. Due to the risk of orbital cellulitis, meningitis, and brain abscess, dacryocystitis must be treated aggressively with 7–10 days of systemic antibiotics with empiric coverage for *Streptococcus* and *Staphylococcus* species (typically vancomycin or clindamycin depending on disease severity) until cultures of blood and nasolacrimal duct fluid can direct targeted therapy [164,168]. 

## 11. Disorders of the Eyelids and Skin

Congenital ptosis is drooping of the eyelid that presents at birth or within 1 year in roughly one in 842 live births [169]. It is usually unilateral and is most frequently due to developmental errors causing infiltration or even replacement of the levator palpebrae superioris muscle with fibrous and adipose tissue [170]. Congenital ptosis can result in amblyopia if the eyelid obscures the pupil or exerts enough pressure on the cornea to alter its morphology and induce astigmatism [171,172]. Treatment is surgical correction and is indicated in cases at risk for amblyopia at any age [170,173]. In cases where amblyopia is not a pressing concern, surgery should be delayed until at least age 3–4 years for improved surgical success [174]. 

Capillary (strawberry) hemangiomas are common, benign vascular tumors that occur in up to 5% of live births [175]. Although they may present anywhere on the skin, mucosa, or internal organs, they most commonly appear on the head and neck and can implicate the eyelid and extend into the orbit [176]. Due to their local compressive effects, they can cause mechanical ptosis, strabismus, and astigmatism, ultimately resulting in amblyopia. Capillary hemangiomas follow a classical disease course, appearing spontaneously at birth or within the following weeks, undergoing a phase of rapid proliferation lasting 5–6 months, proliferating slowly or plateauing until beginning involution around 1 year of age, then resolving completely over several years [177,178]. Due to their predictable course, capillary hemangiomas are initially managed with observation only. However, intervention is indicated when there is a risk for amblyopia, optic nerve compression, and other threats to vision [179]. When indicated, treatment is best initiated as soon as possible, preferably before 4 weeks of age, to stymie the rapid proliferation phase. The first-line treatment is oral propranolol, although systemic corticosteroids can be used as a second-line agent for cases in which propranolol is contraindicated [180,181]. Surgical therapies can also be considered, such as laser photocoagulation for superficial lesions and surgical excision for tumors refractory to first-line therapies [182,183].

Dermoid cysts are benign tumors composed of keratinized epithelial and adnexal components that account for 46% of all childhood orbital neoplasms [184]. They typically present as a smooth, superficial mass near the lateral brow or less frequently medially. Although they are often asymptomatic and sometimes regress spontaneously, they can be slowly progressive and are usually surgically excised before they rupture and cause inflammation [185,186].

## 12. Discussion

Given the importance of vision in both the quality of life and the development of pediatric patients, early screening, diagnosis, and treatment of ocular disease are crucial aspects of their care. Multiple American professional societies, including the AAO, AAP, AAPOS, and USPSTF, support vision screening in children to identify threats to vision and intervene early in their course, thus preventing long-term vision impairment. Of note, no randomized controlled trials have been performed to demonstrate that vision screening programs reduce the incidence of amblyopia in older children or adults, representing an area for future research [187].

Pediatricians performing well-child checks are often the first to detect ocular pathology in children. While less serious problems like conjunctivitis may be managed well by primary care physicians, the AAP recommends referring to a pediatric ophthalmologist for more serious cases such as those with suspected or diagnosed retinoblastoma or other ocular or orbital tumors, cataracts, glaucoma, congenital ocular defects or infections, systemic syndromes or genetic disorders with potential ocular manifestations, abuse with an eye injury, or any suspected eye disease in patients 7 years of age or younger who are nonverbal or cannot read [9]. Parents and guardians also play a key role as they often observe worrying symptoms between routine screenings. They may be educated to bring patients for evaluation if they display symptoms best described in plain language, such as whitening of the pupil (leukocoria), eyes that look crooked or crossed (strabismus), eyes that do not move together (disconjugate gaze), frequent squinting, drooping eyelid (ptosis), seeing double (diplopia), excessive tearing (epiphora), pupils of different sizes (anisocoria), light sensitivity (photophobia), and pain, redness, swelling, crusting, or discharge of the eyes or eyelids for over 24 h.

Screening guidelines, diagnostic criteria, and treatment options for pediatric ocular diseases constantly evolve as new research and innovations improve the standard of care. Therefore, it is important for clinicians working with pediatric populations to maintain an up-to-date understanding of guidelines and recommendations to inform their decision-making as they work to protect the vision of children.

## Figures and Tables

**Table 1:** Pediatric Vision Screening Tests and Referral Indications by Age.

Test	Referral Indications	Birth to 6 Months	6–12 Months	1–3 Years	3–4 Years	4–5 Years	6+ Years
Red reflex	Absent, white, dull, opacified, or asymmetric	•	•	•	•	•	•
External inspection	Structural abnormality (e.g., ptosis)	•	•	•	•	•	•
Pupillary examination	Irregular shape, unequal size, poor or unequal reaction to light	•	•	•	•	•	•
Fix and follow	Failure to fix and follow	Cooperative infant ≥3 months	•	•			
Corneal light reflection	Asymmetric or displaced		•	•	•	•	•
Instrument-based screening	Failure to meet screening criteria			•	•	•	•
Cover test	Refixation movement				•	•	•
Visual acuity	Worse than 20/50 either eye or 2 lines of differences between the eyes				•	•	•
Visual acuity	Worse than 20/40 either eye					•	•
Visual acuity	Worse than 3 of 5 optotypes on 20/30 line, or 2 lines of difference between the eyes						•

Reprinted from *Ophthalmology*, *125*, Wallace DK, Morse CL, Melia M, et al., Pediatric Eye Evaluations Preferred Practice Pattern^®^: I. Vision Screening in the Primary Care and Community Setting; II. Comprehensive Ophthalmic Examination, P184–P227, Copyright 2017, with permission from the American Academy of Ophthalmology. Similar recommendations from the AAP and AAPOS are nearly identical [15]. Guidelines from the USPSTF only recommend vision screening for children of ages 3–5 years [16].

## Data Availability

Not applicable.
